# Internalization of therapeutic antibodies into dendritic cells as a risk factor for immunogenicity

**DOI:** 10.3389/fimmu.2024.1406643

**Published:** 2024-08-28

**Authors:** Michel Siegel, Anna-Lena Bolender, Axel Ducret, Johannes Fraidling, Katharina Hartman, Cary M. Looney, Olivier Rohr, Timothy P. Hickling, Hubert Kettenberger, Martin Lechmann, Céline Marban-Doran, Thomas E. Kraft

**Affiliations:** ^1^ Roche Pharma Research and Early Development, Pharmaceutical Sciences, Roche Innovation Center Basel, Basel, Switzerland; ^2^ Roche Pharma Research and Early Development, Pharmaceutical Sciences, Roche Innovation Center Munich, Penzberg, Germany; ^3^ Unité Propre de Recherche CNRS 9002 RNA, Université de Strasbourg, Strasbourg, France; ^4^ Institut Universitaire de Technologie Louis Pasteur, Université de Strasbourg, Schiltigheim, France

**Keywords:** immunogenicity, biotherapeutics, charge patches, internalization, dendritic cells

## Abstract

**Introduction:**

Immunogenicity, the unwanted immune response triggered by therapeutic antibodies, poses significant challenges in biotherapeutic development. This response can lead to the production of anti-drug antibodies, potentially compromising the efficacy and safety of treatments. The internalization of therapeutic antibodies into dendritic cells (DCs) is a critical factor influencing immunogenicity. Using monoclonal antibodies, with differences in non-specific cellular uptake, as tools to explore the impact on the overall risk of immunogenicity, this study explores how internalization influences peptide presentation and subsequently T cell activation.

**Materials and methods:**

To investigate the impact of antibody internalization on immunogenicity, untargeted toolantibodies with engineered positive or negative charge patches were utilized. Immature monocyte-derived DCs (moDCs), known for their physiologically relevant high endocytic activity, were employed for internalization assays, while mature moDCs were used for MHC-II associated peptide proteomics (MAPPs) assays. In addition to the lysosomal accumulation and peptide presentation, subsequent CD4+ T cell activation has been assessed. Consequently, a known CD4+ T cell epitope from ovalbumin was inserted into the tool antibodies to evaluate T cell activation on a single, shared epitope.

**Results:**

Antibodies with positive charge patches exhibited higher rates of lysosomal accumulation and epitope presentation compared to those with negative charge patches or neutral surface charge. Furthermore, a direct correlation between internalization rate and presentation on MHC-II molecules could be established. To explore the link between internalization, peptide presentation and CD4+ T cell activation, tool antibodies containing the same OVA epitope were used. Previous observations were not altered by the insertion of the OVA epitope and ultimately, an enhanced CD4+ T cell response correlated with increased internalization in DCs and peptide presentation.

**Discussion:**

These findings demonstrate that the biophysical properties of therapeutic antibodies, particularly surface charge, play a crucial role in their internalization into DCs. Antibodies internalized faster and processed by DCs, are also more prone to be presented on their surface leading to a higher risk of triggering an immune response. These insights underscore the importance of considering antibody surface charge and other properties that enhance cellular accumulation during the preclinical development of biotherapeutics to mitigate immunogenicity risks.

## Introduction

Immunogenicity, in this context referring to an unwanted immune response that occurs after the administration of a biotherapeutic and results in the production of anti-drug antibodies (ADAs), represents a significant risk factor for the development of therapeutic antibodies. In many patients, an immune response to a therapeutic antibody leads to diminished efficacy by reducing exposure and/or having a negative impact on its safety profile. As for anti-idiotypic antibodies, it is also important to consider the impact of anti-allotypic antibodies, which may also lead to undesirable effects in patients. Immunogenicity is a multifactorial phenomenon and risk factors influencing each and every step of the immune response may have major overall consequences.

The consequences of high clinical immunogenicity incidence can change the risk-benefit profile of a drug and may lead to the discontinuation of its clinical development. In 2016, the FDA conducted a review of 121 approved biological products and their immunogenicity profile. For the majority of these drugs (89%, n=108) the incidence of immunogenicity was reported and nearly half of these products (n=59) reported an impact on efficacy while 60% (n=73) reported an impact on safety (n = 73) due to immunogenicity (1). Although this analysis does not account for the molecules that were discontinued during clinical development due to negative effects of ADAs, it strikingly exemplifies the need for generating a solid understanding of the root causes of immunogenicity for therapeutic proteins and for engineering strategies to optimize clinical candidates, thereby enhancing the likelihood of successful drug development.

Due to the high sequence diversity of naturally occurring antibodies, a certain proportion of them carries a positive or negative net charge on their isopotential surface as exemplified by the charge distribution of human intravenous immune globulin (IVIG) by Kraft et al. (2). Antibodies, generated for therapeutic use via immunization of animals or via unbiased phage display libraries show similar biophysical properties. A crucial step in the art of antibody drug development is to either exclude molecules during early development that do not fulfill the properties of a potentially successful drug (such as, efficacy, developability, pharmacokinetics, toxicity or immunogenicity) or to skillfully alter the amino acid sequence to improve one property while maintaining the others. In order to do so, we first need to understand the effect that certain biophysical properties have on the drug-like properties of a therapeutic antibody.

Previously, it has been observed that biophysical properties such as positive charge patches affect the biodistribution of therapeutic Abs and pharmacokinetic properties like nonspecific clearance, by altering their uptake into endothelial cells (3–6). We hypothesized that the same biophysical properties affecting the nonspecific pharmacokinetic properties could also affect internalization into dendritic cells (DCs) and thus the risk for immunogenicity. In order to test this hypothesis, we measured the effects of 5 well characterized IgG1-based tool antibodies with a high sequence homology but different biophysical properties (e.g. charge patches) on several key steps in the immunogenicity response cascade.

There are four critical steps in the generation of an adaptive immune response: 1) the internalization and processing of the antigen, followed by 2) its presentation to specific CD4+ T cells, and their activation 3) which, together with co-stimulation will then 4) interact with a B cell inducing their differentiation and the subsequent production of antibodies (Abs). We first explored the influence of internalization rates in DCs and the subsequent cellular accumulation (step 1) of Abs upon initiation of an immunogenic response. The main drivers regulating nonspecific cellular accumulation of Abs are fluid phase endocytosis and recycling via the neonatal Fc receptor (FcRn) (7). Additionally, clathrin-dependent receptor-mediated, and caveolae-mediated endocytosis can also contribute to cellular accumulation of Abs (8) as well Fc-gamma receptors (FcγRs) (9). All of these mechanisms are recapitulated in the DC internalization assay described elsewhere (10), where we measure the rate of accumulation of a compound in the lysosomes of human monocyte-derived DCs. We subsequently determined the presentation of peptides derived from these tool compounds on MHC-II molecules (step 2) and finally assessed the rate of CD4+ T cell activation (step 3) to determine the influence of biophysical properties and subsequent DC accumulation rate on the risk of causing an immunogenic response. T cell precursors, specific to a certain biotherapeutic and capable of eliciting an immunogenic response are extremely rare (in the order of one in a million) (11). We therefore applied a fit-for-purpose strategy, inserting a dominant CD4+ T cell epitope from ovalbumin (12) into our test Abs, thus increasing the likelihood of a specific T cell response directed towards a robust T cell epitope where we can directly compare the impact of internalization and presentation rate of the same epitope on T cell activation.

Here, we propose an integrated approach looking at the consequences of charge patches and altered internalization into monocyte derived DCs (moDCs) for the subsequent steps of the immune response.

## Results

### Positive charge patches enhance the internalization into moDCs.

**Figure 1:**
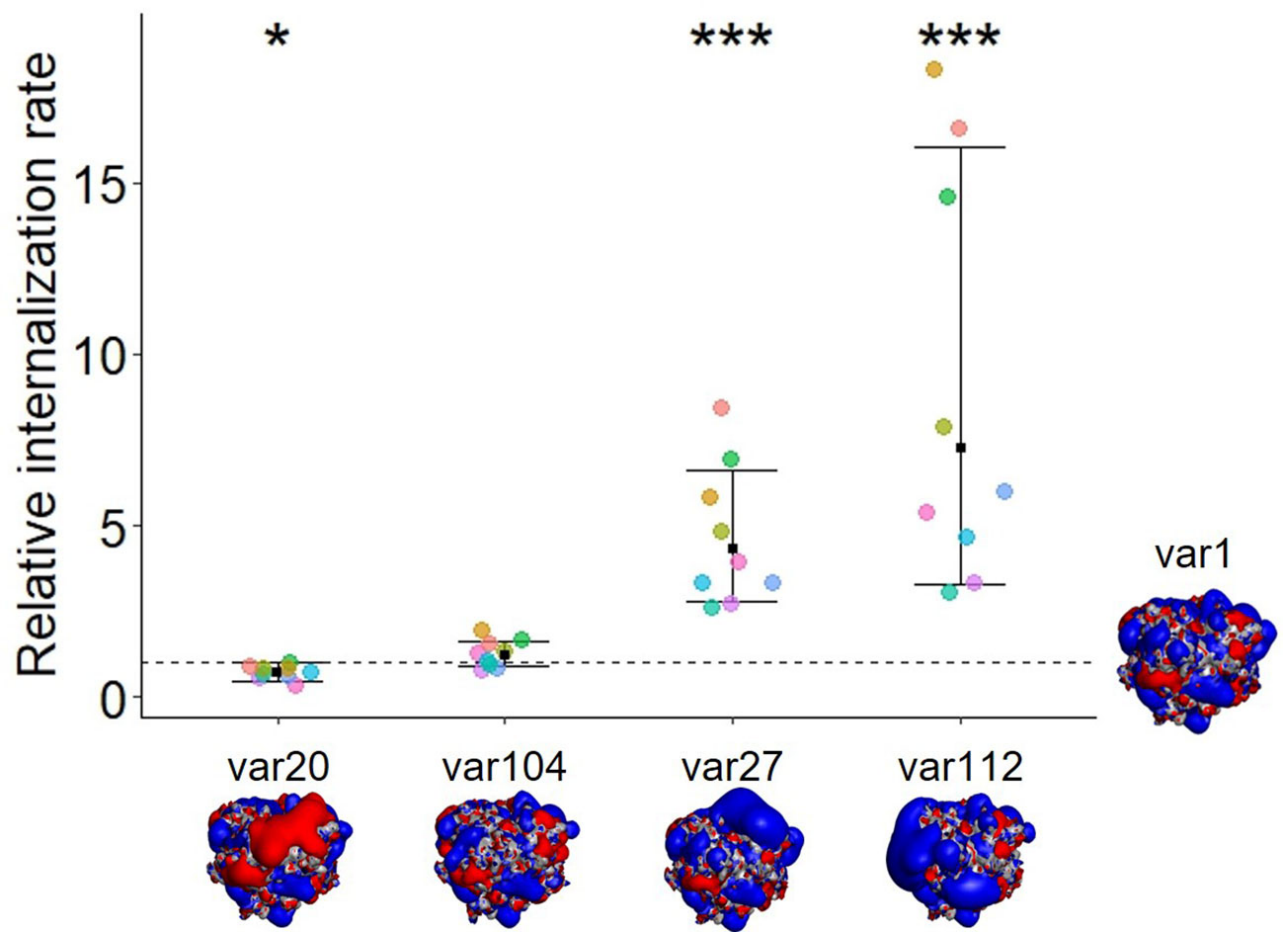
The modification of biophysical properties such as the insertion of charge patches or the modification of charge distribution alters the internalization rate of untargeted antibody variants. The dot plot represents the relative internalization rate of each variant normalized to var1, where the internalization rate of var1 lies at 1 (each color representing a donor, n=9). The internalization rate corresponds to the slope of the mean fluorescence, (gMFI, subtracted for background over time (0, 120 min, normalized to each dosing solution fluorescence). The mean fold change to var1 is displayed for each group along with the 95% confidence interval. A mixed effect model with random donor intercepts on log transformed data has been applied and the effect of changing the antibody sequence (variants) compared to var1 (parental mAb) was assessed by the least squares mean method corrected for multiple comparison (p< 0.001= ***; p< 0.05= *). The isopotential surfaces of one of each antibodies’ Fabs (viewed from the top, looking at the CDR region) are shown (blue: positive charges; red: negative charges, gray neutral). Isocontour renderings shown were generated using Discovery Studio (BIOVIA, Dassault Systèmes, Discovery Studio 2019, version 19.1.0.18217, San Diego: Dassault Systèmes).

All antibody variants described here were derived from a common human IgG1 non-targeted antibody, named variant 1 (var1) with abolished classical Fcɣ receptor interactions (13). Surface-exposed residues were manually identified in the modeled three dimensional structure of the variable domain and replaced with selected amino acids carrying specific properties like positive, negative or neutral net charge. Specifically, surface exposed amino acid residues in the antibody variable fragment (Fv region) were selected to create molecules with high sequence similarity but distinct biophysical properties like charge patches and charge distribution (**Figure 1**). The resulting variants represent distinct characteristics like positive charge patches (var27 - positive charge patch on heavy chain; var112 - positive charge patch on light chain), negative charge patches (var20 - negative charge patch on heavy chain) or even charge distribution (var104). These molecules were designed and produced together with the parental mAb (var1). The parent molecule and the four variants exhibit significant differences in their calculated isoelectric points (from 4.8 for var20 up to 9.8 for var112) correlating with the substantial difference in the number of charged residues in the Fv domain.

The presence of positive charge patches on an mAb was found to enhance tumor and organ uptake (3, 4) as well as uptake into Madin-Darby canine kidney cells (14) and human primary endothelial cells (internal, unpublished data). In order to determine whether this observation would translate to other cell types, specifically relevant to immunogenicity, we investigated the cellular accumulation rate of these five antibody variants into moDCs (**Figure 1**). Immature moDCs were used in this study to recapitulate their physiological characteristics to internalize antigens or pathogens at a high rate (15, 16). In order to compare the cellular internalization and accumulation rates of the different antibody variants, we used the DC internalization assay (DCIA) (10) where we labeled the molecules with a pH-sensitive fluorophore and measured the cellular fluorescence using flow cytometry after incubating the moDCs for two hours with the labeled compounds (see Material and Methods and Equations 1, 2). Results from the DCIA show a significantly higher cellular accumulation rate into moDCs for the two antibodies containing positive charge patches (var27 and var112) compared to the parental molecule (var1), the one engineered with a negative charge patch (var20) and the one with an even charge distribution (var104). Var20 showed a small but statistically significant decrease in cellular accumulation rate. These observations point towards a correlation between the presence of positive charge patches and an increased rate of internalization in moDCs, which may potentially increase the risk of initiating an immunogenic response.

### Increased internalization enhances T cell epitope presentation by moDCs

**Figure 2:**
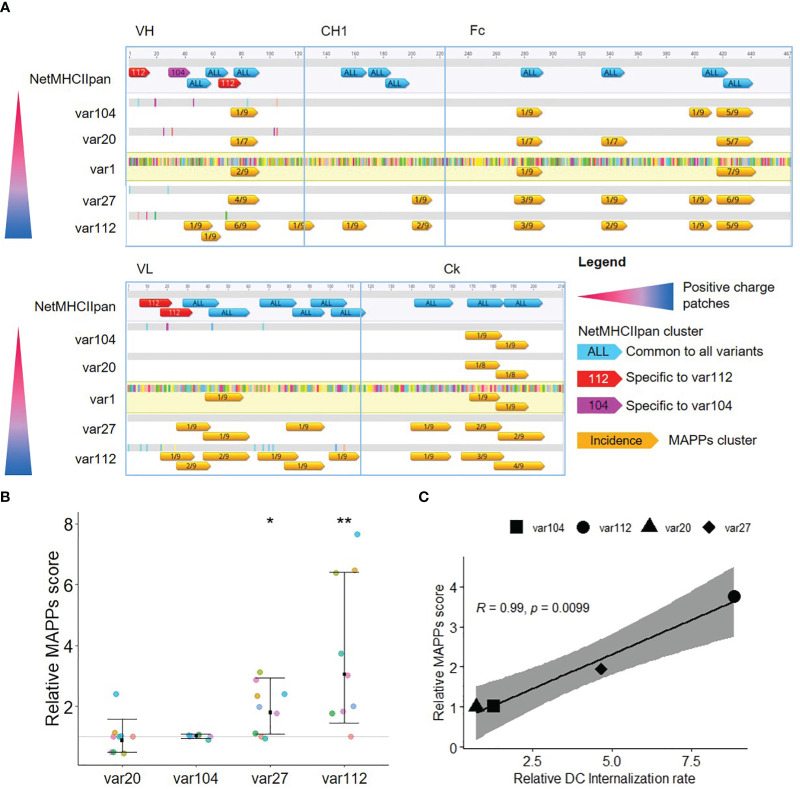
Increased cellular accumulation rate leads to increased peptide presentation. **(A)** Analysis of the T cell epitope content for the five antibody variants. The differences in amino acids to var1 are highlighted (color corresponding to the amino acid). Epitopes detected by MAPPs (in orange, annotated with the proportion of donors presenting the T cell epitope) are represented together with T cell epitope prediction (annotated with the number of strong binders, SB) along the amino acid sequence. T cell epitope predictions in blue are common to all variants whereas the ones in pink and red are respectively specific for var104 and var112. **(B)** Comparison of the MAPPs score for the different antibody variants. The MAPPs score summarizes the number of epitopes detected and their signal intensities (nepitopes x mean(log2(signal)) normalized to var1 (each color representing a donor, n=9). The mean fold change to var1 is displayed for each group along with the 95% confidence interval. A mixed effect model with random donor intercepts on log transformed data has been applied and the effect of changing the antibody sequence (variants) compared to var1 (parental mAb) was assessed by the least squares mean method corrected for multiple comparison (p< 0.01= **; p< 0.05= *). **(C)** Correlation plot of the MAPPs score according to the DC internalization rate. Relative MAPPs score and DC internalization rate were obtained by taking the mean of all the tested donors (n=9). Pearson’s correlation was used and the 95% confidence interval is displayed.

Internalization and processing of antigens by professional antigen presenting cells (APCs) mainly result in presentation of antigen-derived peptides via MHC-II molecules. However, the relationship between cellular accumulation rates in APCs and presentation of antigen-derived peptides is unclear. In order to explore further this hypothesis, the presentation of T cell epitopes for the five mAb variants was assessed using MHC-II Associated Peptide Proteomics (MAPPs) on cells isolated from the same nine donors previously tested in the DCIA (**Figure 2A**).

**Figure 3:**
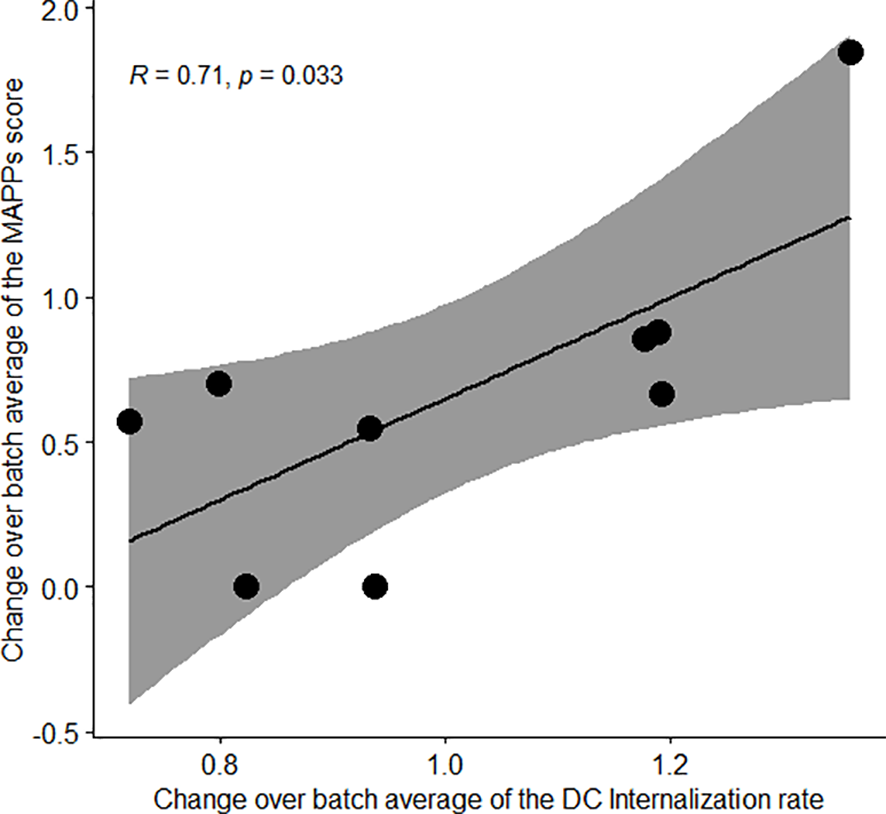
A donor propensity for faster cellular accumulation leads to an increased peptide presentation in MAPPs. Correlation plot of the MAPPs score according to the DC internalization rate. The change over batch average was obtained by taking the mean across all the 5 tested antibodies within a donor, divided by the average across all the donors and antibodies tested in the corresponding batch. Pearson’s correlation was used and the 95% confidence interval is displayed.

All MAPPs-detected sequence clusters were predicted as strong binders using NetMHCII-pan4.0 with the exception for two clusters located in the CH1 domain. The prediction scores for the likelihood of a peptide to be naturally presented by a MHC II molecule. The output is normalized to the prediction of a set of random peptides, and only the top 2% score (for at least one of the 13 DRB1 alleles selected)of this dataset is considered a “strong binder”. This analysis indicates that the residue exchange required to generate the charge patches resulted in no (var27 and var20), one (var104), or four (var112) neo-epitopes in comparison to var1. Most interestingly, we observed a sharp increase in the number of clusters and relative abundance of detected peptides in the MAPPs assay performed with the mAb variants bearing positive charge patches (var27 and var112), specifically for sequence clusters that are conserved between all variants. In particular, the amino acid sequence of the two aforementioned CH1 clusters was present in all tested variants but these clusters were only detected in the MAPPs assay using the two mAb variants with positive patches. To explore this further, we converted the MAPPs data into a score considering, for each mAb variant, the number and intensity of the detected epitopes. This “MAPPs score” shows a clear positive trend for var27 and var112 (**Figure 2B**) that correlates strongly with the relative internalization rate as measured by the DCIA (**Figure 2C**). During the development of the DCIA, we observed that by normalizing the internalization rates to an internal control (var1), we could account for the donor to donor variability (10). This prompted us to hypothesize that the internalization rate could be an intrinsic property of each donor which is correlated with the amount of presented peptides on MHC-II complexes. In order to test this hypothesis, we calculated the fold-change from the average internalization rate and MAPPS score across all tested compounds for each donor. The analysis revealed a clear correlation between both parameters (R of 0.71, p=0.033) supporting the hypothesis that the internalization rate of a donor and the amount of peptide presentation are directly correlated (**Figure 3**).

In summary, these results suggest that Abs with positive charge patches accumulate in the lysosome of moDCs at a higher rate resulting in a generally enhanced presentation of CD4+ T cell epitopes on MHC-II complexes. In contrast, Abs with negative charge patches or an even charge distribution show low cellular accumulation rates and a low to moderate presentation of CD4+ T cell epitopes on MHC-II complexes.

Of note, the relative internalization rate difference between var20 and var1 is considered small (decreased by 30%), and similar scale observations (increased by 30%) in var104 did not achieve statistical significance (likely owing to greater donor variability). As the impact of positive charge patches are much larger and the small difference between var20 and the parental mAb in internalization did not translate to peptide presentation (MAPPs score), the focus was put on the impact of positive charges.

### Increased internalization leads to higher risk of T cell activation

The next step in the immunological response ultimately leading to ADA formation, is the recognition of MHC-II presented peptides by specific CD4+ T cells causing their activation and subsequent expansion. In order to assess whether increased cellular accumulation and presentation of a mAb by DCs would lead to an increase in the specific CD4+ T cell response, we determined the effect on CD4+ T cell proliferation of two of the previously described mAb variants, one with low internalization rate (var1) and one with high internalization rate (var112).

### Insertion of an ovalbumin CD4+ T cell epitope within the antibody variant sequences had no relevant influence on the studied properties

**Figure 4:**
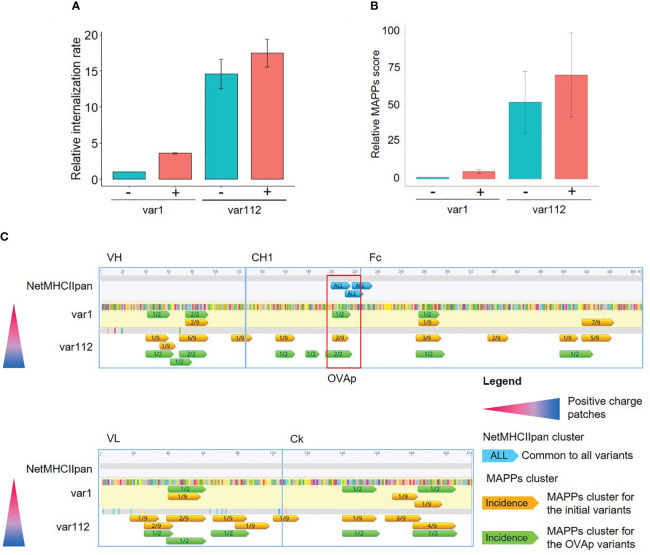
Generation and characterization of ovalbumin CD4+ T cell epitope (OVAp) containing variants. **(A)** Bar plot representing the relative internalization rates of mAb variants before (-) and after (+) the insertion of the OVAp (n=2, 95% confidence interval). **(B)** Bar plot representing the relative MAPPs score of mAb variants before (-) and after (+) the insertion of the OVAp (n=2, 95% confidence interval). **(C)** Analysis of the T cell epitope content of var1 and var112. The differences in amino acids to var1 are highlighted (color corresponding to the amino acid). Epitopes detected by MAPPs (annotated with the proportion of donors presenting the T cell epitope) of the initial variants (in orange) are represented together with the epitopes detected by MAPPs for the OVAp variants (in green) along the amino acid sequence. Only the predicted T cell epitopes induced by the insertion of the OVAp are represented (in blue, common to all variants).

To avoid a bias from sequence differences of the tested Abs, we inserted a strong T cell epitope, the immunogenic peptide from ovalbumin (amino acids 323–339), into the sequence of the test Abs. Similar as already described for IgG3 (17), we show here that IgG1s can tolerate the insertion of a peptide sequence as substitution of the sixth loop (KPSN sequence) in the CH1 domain, as this domain is conserved between both isotypes. As a first step, we tested this new set of tool compounds in the DCIA and the MAPPs assays, to better understand the consequence(s) of inserting the peptide sequence corresponding to the epitope in the tool compounds (**Figure 4**).

The insertion of the OVA CD4+ T cell epitope (OVAp) led to a slight increase in moDCs internalization rates for both compounds (**Figure 4A**). However, differences between mAb variants within the plus or minus OVAp series remain conserved, allowing the qualitative comparison within the molecule series. The same tendency was observed for the MAPPs score (**Figure 4B**) and for the clusters detected in MAPPs (**Figure 4C**): they generally overlap for antibody variants with or without the OVAp insertion. Overall, the data supports the correlation between an increased internalization rate (caused by differences in biophysical properties such as charge) and an enhanced CD4+ T cell epitope presentation via MHC-II, which persists across all variants after insertion of the OVAp. In addition, we confirmed that the OVAp insertion is predicted as a robust T cell epitope, as indicated by NetMHCII-pan4.0 (**Figure 4C** in blue). This sequence was also presented on MHC-II molecules in the MAPPs assay with the OVAp-containing variants (**Figure 4C**, annotated in green and marked in the red box).

### Exposure of DCs to positively charged mAb increases specific CD4+ T cell activation compared to parental mAb

**Figure 5:**
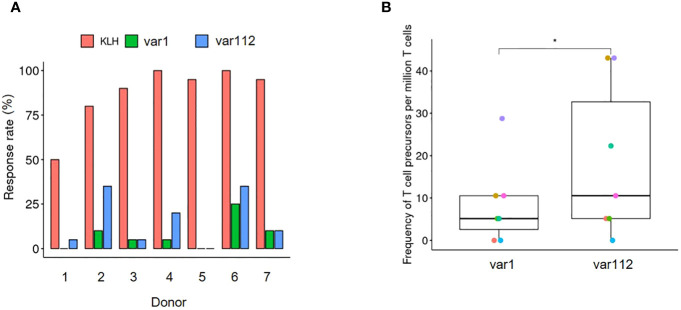
Increased CD4+ T cell activation following increased internalization and peptide presentation directed towards a common epitope. **(A)** Representation of the assay response rate (n= 20) from 7 blood donors according to the treatment. The proportion of positive wells are shown in green, blue and red bars. As positive control T cells have been expanded using autologous moDCs incubated with KLH and their response has been assessed at week 4 by IFNy ELISPOT (red). For the antibody variants, T cells have been expanded using autologous moDCs incubated with var1 (green) or var112 (blue) and their response to autologous moDCs incubated with OVA has been assessed at week 4 by IFNy ELISPOT (see Material and Methods). **(B)** Estimation of the number of T cell precursors for the two tested variants. The calculation has been done according to Delluc et al., 2011. In short the Frequency = -Ln (negative wells/total wells tested)/(CD4 T cells/well)). A one-sided paired T-test has been performed (p< 0.05= *).

We evaluated the relationship between the occurrence of positive surface charge patches on mAbs and their potential to activate CD4+ T cells, the next biological step in the immunological response, for var1 and var112 containing the OVAp. It has been demonstrated that the T cell precursors, capable of expanding in response to a therapeutic mAb, are in the order of magnitude of one specific T cell out of 1 million T cells (11). Therefore, the number of T cells in a T cell assay represents a limiting factor that could potentially be overcome by conducting additional rounds of stimulation, thus selectively expanding specific T cells. This approach has been shown successful elsewhere (18, 19) and when coupling it to the OVAp containing mAb variants, it becomes a suitable strategy for exploring the relationship between internalization, epitope presentation by APCs and CD4+ T cell activation, ultimately leading to immunogenicity. Moreover, the assay format and the insertion of a robust CD4+ T cell epitope within the sequence of the two mAb variants allowed us to focus on this specificity and rule out other confounding factors like sequence differences. The data comparing the CD4+ T cell activation propensity of var1 to var112 are summarized in **Figure 5**.

The proportions shown in **Figure 5A**, represent the number of positive wells out of the 20 initial DC: CD4+ T cell co-cultures (see Material and Methods section). KLH was used as a positive control for its well-documented capacity to induce a strong CD4+ T cell response. The two test mAbs, namely var1 and var112, were evaluated for their propensity to activate CD4+ T cells in co-culture between moDCs incubated with ovalbumin (in this context, refers to the process of internalization and processing) and CD4+ T cells. Out of the 20 co-cultures per donor, cultured over 21 days with weekly re-stimulation by moDCsincubated either with var1-OVAp (green) or var112-OVAp (blue), the ones challenged with var112-OVAp (containing the positive charge patches and showing higher cellular accumulation in moDCs) were more prone to respond to moDCs incubated with ovalbumin. This finding was confirmed by the estimation of the respective CD4+ T cell precursors using the Poisson distribution (**Figure 5B**). Indeed, we determined a significantly higher number of CD4+ T cell precursors for var112-OVAp responsive to ovalbumin, following weekly challenges with the mAb variant. This finding is consistent with earlier observations and connects the increased accumulation of Abs in the lysosomes of moDCs to the increased presentation of CD4+ T cell epitope via MHC-II and subsequently to a higher probability of eliciting an effective CD4+ T cell response.

## Discussion

Several preclinical studies, encompassing *in vivo*, cellular and mechanistic experiments, have investigated the structure-pharmacokinetic relationship of therapeutic antibodies. These studies have highlighted the influence of positive charges on pharmacokinetic parameters, such as faster nonspecific clearance and decreased terminal half-life (4, 5, 7). Since the primary mechanism of nonspecific clearance involves lysosomal degradation in cells with a high endocytosis rate, the accumulation rate in these cells becomes a pivotal determinant impacted directly by the biophysical properties, particularly the charge, of therapeutic antibodies.

Demonstrating this relationship in humans beyond preclinical studies has proven challenging, as therapeutic antibodies with fast clearance often don’t progress to clinical trials. Nevertheless, examples such as briakinumab or boccocizumab demonstrate that a short half-life could be attributed to positive charge patches (7, 20, 21). Establishing a clear structure-immunogenicity relationship is even more challenging due to the multifactorial nature of immunogenicity, varying immunogenic responses among patients, and often unknown root causes of clinically observed immunogenicity for a specific molecule. Therefore, considering antibody features, including biophysical properties, variations in sequence, or potential T-cell epitopes, as risk factors during the preclinical development of therapeutic antibodies is crucial. Consequently, improving knowledge about the immunogenic risk factors based on the structure or biophysical properties of therapeutic antibodies can positively guide the selection and engineering processes of clinical candidates.

In this context, our investigation focused on the impact of antibody surface charge on the risk of an immunogenic response in humans through systematically engineered tool compounds, mechanistic assays mimicking key processes of immunogenicity *in vitro*, and utilizing primary human cells from several human healthy donors. We established a direct link between the cellular accumulation rate of a therapeutic antibody in dendritic cells, the rate of presentation of corresponding peptides on MHC-II complexes, and the rate of specific activation of T-cells. Here, we specifically used immature DCs for the internalization assay as they are predominantly found in peripheral and lymphoid tissues. These cells are characterized by their high rate of endocytosis and low levels of surface MHC-II and costimulators, with their primary function being the capture of antigens. Consequently, we used mature DCs for the MAPPs assay, given that these cells reside mainly in lymphoid tissues, exhibit lower endocytosis rates and higher expression of surface MHC-II and costimulatory molecules, reflecting their main role in the activation of T cells (4).

In the context of cellular accumulation and peptide presentation mechanisms, both nonspecific and target-mediated internalization contribute to an increased rate of cellular accumulation, while in the neonatal Fc receptor (FcRn)-expressing cells, recycling typically leads to a decrease of Fc-containing antibody. Increased internalization of charged antibodies into cells, and subsequently increased lysosomal accumulation, is primarily mediated by nonspecific endocytosis (fluid-phase pinocytosis) (22, 23). Furthermore, internalization of charged proteins induces the accumulation of transferrin and endosomal maturation (24). These findings suggest that increased processing of charged proteins could potentially alter the profile of epitopes presented via MHC-II. Here, we demonstrated that increased internalization of positively charged antibodies alters the pattern of peptide presentation, resulting in a greater number of epitopes being displayed on MHC-II molecules compared to non-charged variants. This represents a significant challenge in antibody development, as the immunogenic potential of a therapeutic antibody is linked to the ability to present CD4+ T cell epitopes via MHC-II (25).

In this study, we used compounds with distinct charge patch profiles as tools to modulate their cellular accumulation rate in order to investigate immunogenicity as a consequence of increased cellular accumulation of antibodies into DCs. We did not find evidence that the positively charged amino acids themselves constitute a risk factor for immunogenicity. Instead, the actual risk factor appears to be the influence these amino acids have on increasing the rate of internalization. This implies that any mechanisms that lead to increased cellular uptake, such as receptor-mediated internalization into dendritic cells (DCs) or activation of DCs by the therapeutic protein, could also contribute to the risk of immunogenicity. Investigating this hypothesis would be worthwhile. Of note, we tested two compounds carrying a positively charged patch on either the heavy chain or light chain of the Fab (var27-HC, var112-LC) and did observe differences in the degree of internalization and MAPPs score. Although the impact of the location of charges on the three dimensional surface of the Fab region is an important question as well, it goes beyond the scope of this manuscript and would need further investigations with a larger set of tool compounds with systematically sampled location of comparable charge patches.

In conclusion, increased internalization rates into dendritic cells, mediated by positive charge patches, emerge as an important risk factor for immunogenicity. We showed that this effect could be captured via both the DCIA and the MAPPs assay and also translates into an increased likelihood of eliciting a CD4+ T cell response. Our findings emphasize the need for a preclinical risk assessment through an integrated approach of assays that capture mechanisms that increase internalization and presentation of lead compounds, thus potentially raising the risk of immunogenicity. In addition, our findings stress the importance of screening out molecules with a high risk of immunogenicity during the early phases of drug development. They also present an opportunity for charge-engineering of lead candidates that were flagged by the aforementioned assays by replacing positively charged amino acids with neutral or negatively charged ones as well as balancing the charges across the surface of the antibody variable domain.

## Materials and methods

### Design of antibody surface charge variants

Using an existing human IgG1 antibody with abolished binding to its original target and containing an FcyR silencing mutation (PG LALA), as template (var1), we replaced individual surface exposed amino acids in the variable Fragment to create variants with specific biophysical properties (pIvar1 = 9.49, pIvar20 = 7.81, pIvar27 = 10.02, pIvar104 = 8.96, pIvar112 = 10.16). Isopotential point (pI) was calculated based on the modeled structures of the antibody variants. The amino acid sequences of the variable domain VH were used to create a homology model using the MoFvAb software version 7 (Bujotzek et al., 2015). By using an in silico calculation method starting with the homology model, followed by pH- protonation of acidic and basic side-chains between pH 2 to pH 12 with pH steps of 0.1, we calculated the isoelectric points using the software CHARMM and Delphi as implemented in the software suite Discovery Studio (vendor: Dassault Systems).

Mutation sites were both CDR and framework residues. Sequence details are shown in **Supplementary Figure 1** and isopotential surfaces in **Figure 1**.

The initial variants (var20, var104, var1, var27, var112) were further engineered by inserting the main CD4+ T cell epitope derived from ovalbumin into the sixth loop of the C(H)1 domain (12). Specifically, the loop sequence KPSN in CH1 was replaced by the OVA sequence ISQAVHAAHAEINEAGR.

### Protein preparation and analytics

Monoclonal antibody variants were produced in house using standard expression and purification protocols. HEK293 cells were transiently transfected and antibodies in supernatant were purified via protein A column and preparative size exclusion chromatography (SEC). Antibodies were confirmed to be >95% monomeric via analytical SEC, correct chain pairing ratio was determined via capillary electrophoresis and the identity was confirmed via electrospray ionization—mass spectrometry (ESI-MS).

### Proteins

Stock solutions of keyhole limpet hemocyanin (KLH-Imject Maleimide-Activated mcKLH, Thermo Fisher Scientific, #77600) were reconstituted and stored at -80°C in single-use aliquots according to the manufacturer’s recommendations under sterile conditions.

### Antibody labeling

For the DC internalization assay, antibodies were labeled using the SiteClick Antibody Azido Modification Kit (Thermo Fisher, #S20026) according to the manufacturer’s instructions. Briefly, terminal N-linked galactose residues on the glycosylation of the Fc region were removed by β-galactosidase and replaced by an azide-containing galactose via the β-1,4-galactosyltransferase. This azide modification enables a copper-free conjugation of sDIBO-modified dyes. Here, the pH-sensitive amine-reactive fluorophore (pHAb, Promega, #G9841) was coupled to a sulfo-DBCO PEG4 amine and conjugated to the azide-modified antibody. Antibodies were labeled with a molar dye excess of 3.5–4. Excess dye was removed using the Amicon Ultra-2 Centrifugal Filter (Merck, #UFC205024) with a MWCO of 50 kD and antibodies were re-buffered in 20 mM histidine 140 mM NaCl buffer (pH 5.5). The fluorescence of the dosing solution was measured in a Tecan Infinite Pro 300 fluorometer. 50µl of dosing solution was mixed with 150µl citric acid buffer (0.2 M Citrate-Phosphate buffer pH 4.5) and the fluorescence was measured with an excitation at 532 nm and emission at 560 nm. The absorbances of the labeled molecules at 280 nm and 532 nm were determined using a Nanodrop spectrometer and the concentration [1] as well as the dye-to-antibody ratio (DAR) [2] was calculated as follows:


(1) [1] c(AB)=[A280nm−[A280nm*CF(Dye)]]/ϵ(AB)

(2) [2] DAR=[A532nm*MW(AB)]/[c(AB)*ϵ(Dye)]

(A = absorbance; AB = antibody; c = concentration; DAR = dye to antibody ratio; ϵ (dye) = extinction coefficient dye = 47225; CF = correction factor = 0.36).

### Quality control of the labeled antibodies

To confirm the efficient removal of unbound dye and the absence of possible antibody aggregates or fragments, a size exclusion chromatography of the labeled antibodies and their unlabeled counterparts was performed. Samples were separated using a BioSuite Diol (OH) column (Waters, 186002165) with a potassium dihydrogen phosphate buffer (pH 6.2) as the mobile phase at a flow rate of 0.5 ml/min. Detectors at 280 nm and 532 nm were used to quantify and analyze the labeled antibodies.

### Cell culture and maintenance

Human peripheral blood mononuclear cells were isolated by Ficoll density gradient centrifugation from buffy coats (blood donation center in Aarau, Switzerland) according to the manufacturer’s instructions (GE Healthcare #17–1440-03). For further enrichment of monocytes, magnetic activated cell sorting was performed using anti-huCD14 beads (Miltenyi, #130–050-201) and LS columns (Miltenyi, #130–042-401) according to the manufacturer’s instructions. Briefly, monocytes and beads were incubated in MACS Buffer for 15 min on ice and separated by a magnet. The isolated monocytes were suspended in a pre-warmed medium. CD14+ monocytes were differentiated into monocyte derived DCs (moDCs), by culturing within a DC medium (sterile filtered CellGenix GMP DC medium, with GlutaMAX, non-essential amino acids, sodium pyruvate and Penicillin-Streptomycin) supplemented with 5 ng/mL rhIL4 (R&D systems, #204-IL) and 50 ng/mL rhGM-CSF (R&D system, #215GM-500) for 5 days at 37°C and 5% CO_2_ ambient on ultra-low attachment culture dishes (0.3x10^6^ cells/ml, Corning, #354407). Cells from the same donors were used for both the DCIA and the MAPPs assay in order to better compare the results.

### Dendritic cell internalisation assay

On the day of the experiment, cells were detached from the ultra-low attachment culture dishes by pipetting and plated into ultra-low attachment 96-well plates at a density of 8x10^4^ cells/well (50µl/well). Antibody dosing solutions were prepared at a concentration of 400 nM in DC medium at pH 7.4 and 50 µl were applied to the cells for a final concentration of 200 nM. Cells were incubated for two and four hours at 37°C and 5% CO_2_. Cells were transferred into U-bottom 96-well plates for sedimentation (300 g, 5 min), the pellet was washed with 200 µl ice cold PBS, centrifuged and resuspended in 200 µl FACS buffer containing 50 ng/mL DAPI. The geometric mean fluorescent intensity (gMFI) of the internalized antibodies per cell was acquired using a Fortessa X20 flow cytometer (BD) equipped with a 532 nm-emitting laser. Signals were collected at 572 nm ± 35 nm. The same conditions, gains, and gates were used for all data points. Data extraction was performed using the FlowJo-V10.8.1 software (BD Life Sciences). Cells were gated for singlets, morphology and viability. Geometric mean fluorescent intensity values for the cell population of the negative control (background fluorescence of mock treated cells) were subtracted from gMFI of the cells incubated with the test antibodies of the corresponding donors, followed by normalization to the fluorescence of the antibody dosing solution in order to account for differences in labeling efficiency between antibodies. The fluorescence intensity of the dosing solution was measured with a Tecan Infinite M PLEX instrument. The labeled molecules were prepared in medium for the incubation timespoints at the desired concentration of 200 nM. This dosing solution was diluted in a 1:3 ratio with a 0.2M citric acid buffer (pH 4.5). Fluorescence detection was carried out using an excitation wavelength of 532 nm and an emission wavelength of 560 nm. The normalized geo-mean values from each antibody were plotted against time and fitted via a linear regression curve using R Statistical Software (v4.1.2; R Core Team 2021) to extract the slope (gMFI/min for 120 min). In order to compare donors to each other, slopes of different variants were divided by the slope of var1 to yield the relative internalization rate.

### MHC-II associated peptide proteomics

MAPPs assay was performed according to the standard protocol and analyzed according to Steiner et al. (26). In short, moDCs cells were challenged with the test protein at 300 nM in the presence of 1 μg/mL of lipopolysaccharide (LPS) from Salmonella abortus equi (Sigma-Aldrich Chemie GmbH, Buchs, Switzerland) for 24 h. Mature moDCs were harvested, washed with PBS and the cell pellets were frozen at −80°C. Frozen cell pellets were lysed in 20 mM Tris-buffer solution pH 7.8 containing 1% (v/v) Digitonin and protease inhibitors (Roche Diagnostics GmbH, Mannheim, Germany) for 1 h at 4°C on a ThermoMixer at 1100 rpm. The HLA-DR immune complexes were isolated by immunoprecipitation using the anti human HLA-DR monoclonal antibodies (Clone L243, BioLegends) coupled with NHS-activated magnetic Sepharose beads (GE Healthcare Europe, GmbH). Lysates were incubated with the Sepharose beads on a shaker on a rotator overnight at 4°C. Samples were washed five times with a buffer containing 20 mM N-(2-hydroxyethyl)piperazine-N′-ethanesulfonic acid-NaOH (pH 7.9), 150 mM KCl, 1 mM MgCl2, 0.2 mM CaCl2, 0.2 mM ethylenediaminetetraacetate, 10% (v/v) glycerol, and 0.1% (v/v) Digitonin and five times with purified water. MHC-II peptides were eluted twice from HLA-DR molecules by adding 18 μL of 0.1% trifluoroacetic acid. The eluates were collected and analyzed by tandem mass spectrometry. Detected peptides were grouped into clusters and represented along the sequence of the corresponding antibody using Geneious Prime 2022.1.1 (https://www.geneious.com). A numerical estimation of the MAPPs assay outcome was calculated using the number of epitopes detected and their signal intensities like follows:


$$n_{epitopes\;}x\;mean\left(log_{2}\left(signal\right)\right)$$

### *In silico* T cell epitope prediction

NetMHCIIpan-4.0 (29) was used to predict potential T cell epitope content for the five antibody variants. The algorithm has been run for 13 DRB1 alleles (DRB1–0101, DRB1–0301, DRB1–0401, DRB1–0701, DRB1–0801, DRB1–0901, DRB1–1001, DRB1–1101, DRB1–1201, DRB1–1301, DRB1–1401, DRB1–1501, DRB1–1601) screening for binding affinities of 15-mer derived from the antibody sequences. The top 2% of the hits for at least one of the 13 DRB1 alleles, were kept as strong binders and further analyzed. Detected peptides were grouped into clusters and represented along the sequence of the corresponding antibody using Geneious Prime 2022.1.1 (https://www.geneious.com). For the estimation of the T cell precursor frequency, spots were counted in a computer-assisted video image analyzer (AID, Strassber, Germany). A response was considered positive when the spot count was increased by 2-fold compared to the well where only moDCs were added. The frequency of CD4+ T cell precursors was calculated as proposed in (11), using the Poisson distribution:


Frequency=−ln(negative wells/total wells tested)/(CD4 T cells/well)

Statistical significance was calculated using a one-sided T-test. Statistical analysis was performed using R (v4.1.2; R Core Team 2021).

### Estimation of the T cell precursor frequency

This assay has been performed according to the published protocol proposed by Delluc et al. (11). In short, moDCs were incubated either with KLH, var1 or var112 (300 nM) and matured over night with 1 ug/mL of LPS (Sigma-Aldrich #L5886). T cells were isolated from PBMCs by negative selection (Myltenyi, #130–096-533) as recommended by the manufacturer and co-cultured with autologous moDCs. 10,000 moDCs were co-cultured with 100,000 T cells (n=20 wells per condition) in a total volume of 200 uL of Iscove’s Modified Dulbecco’s Medium (IMDM, GIBCO #21980–032) supplemented with 10% human serum (Sigma-Aldrich #H3667–100ml), 1000 U/mL rh-IL-6 (R&D Systems #7270-IL-025/CF), 10 ng/mL rh-IL-12 (R&D Systems #10018-IL-020) and incubated at 37°C and 5% CO_2_ ambient on ultra-low attachment 96-well plates (Corning #3474). The CD4+ T cells were re-stimulated on day 7 and 14 with fresh autologous moDCs incubated with one of the antibody variants or KLH, 10 U/mL of IL-2 (R&D Systems #202-IL-010/CF) and 5 ng/mL of IL-7 (R&D Systems #207-IL-010/CF). At day 21, the specificity of the CD4+ T cells was assessed by IFN-γ enzyme-linked immunospot (ELISpot) following the recommended procedure (Mabtech, #3420–4APT-10). To assess the T cell response against KLH, 3300 moDCs (either naïve or incubated with KLH 4 hours prior co-culture without LPS) were added to 10,000 CD4+ T cells (n=20) expanded for KLH. The T cell response directed against the two antibody variants was measured in relation to a single epitope (the one presented by the OVA peptide): 3300 moDCs (either naïve or incubated with ovalbumin (Fisher Scientific #A/1280/48) 4 hours prior co-culture without LPS) were added to 10,000 CD4+ T cells (n=20) expanded for either var1 or var112 containing the main ovalbumin CD4+ T cell epitope as introduced earlier. Each of these co-cultures were evaluated in duplicate. A positive value was assigned to culture wells with a spot count that was 2-fold higher in the presence of the protein (ovalbumin or KLH) than in its absence (untreated control). The proportion of positive wells over the total number of wells (n=20) is then used to evaluate the response.

## Data Availability

The raw and processed mass spectrometric data have been deposited to the PRIDE archive (https:proteomecentral.proteomexchange.org/cgi/GetDataset?ID=PXD055197) via the MassIVE partner repository (MassIVE dataset identifier: MSV000095695).

## Supplementary material

Supplementary Figure 1 Sequence alignment for the antibody variants. The sequence of the heavy chain **(A)** and light chain **(B)** are aligned and annotated with the corresponding CDR regions in yellow.

Supplementary Figure 2 Schematic representation of the experimental procedure for the expansion and specificity determination of T cell lines *in vitro*. T cells have been expanded using autologous var1, var112 or moDCs incubated with KLH and their response to autologous moDCs incubated with OVA or KLH (positive control) has been assessed at week 4 by IFNy ELISPOT. The protocol has been adapted from (11) and the figure was created in BioRender.com.
