# Supplementary figures and images for: Internalization of therapeutic antibodies into dendritic cells as a risk factor for immunogenicity
Source: Front Immunol. 2024 Aug 28;15:1406643. doi: 10.3389/fimmu.2024.1406643 (PMC11387504; doi:10.3389/fimmu.2024.1406643)

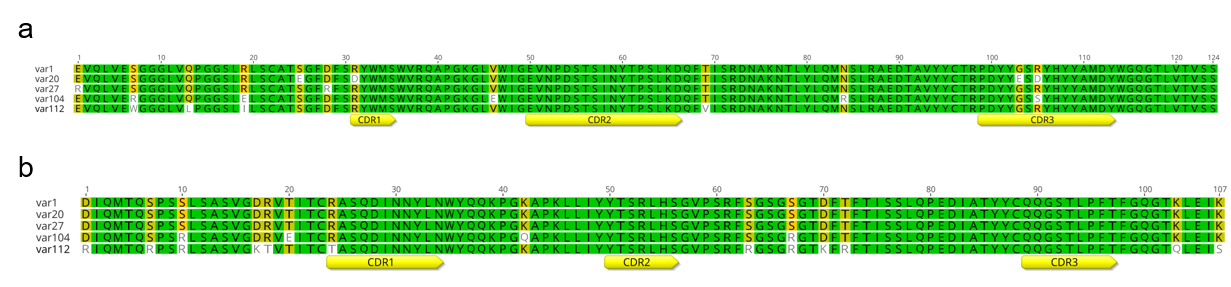

Supplement: Supplementary Figure 1 — Sequence alignment for the antibody variants. The sequence of the heavy chain (A) and light chain (B) are aligned and annotated with the corresponding CDR regions in yellow. [file Image_1.jpeg]

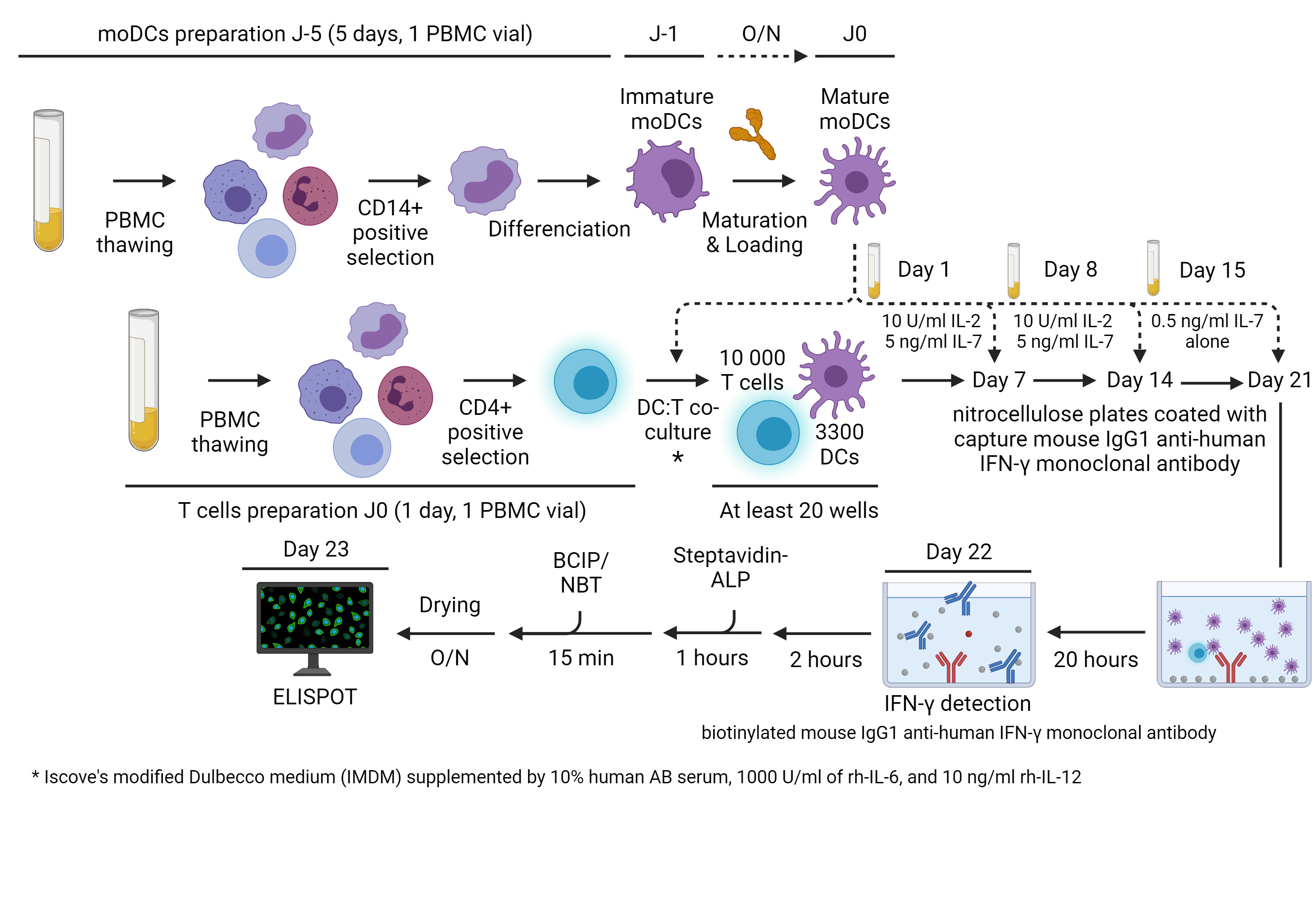

Supplement: Supplementary Figure 2 — Schematic representation of the experimental procedure for the expansion and specificity determination of T cell lines in vitro. T cells have been expanded using autologous var1, var112 or moDCs incubated with KLH and their response to autologous moDCs incubated with OVA or KLH (positive control) has been assessed at week 4 by IFNy ELISPOT. The protocol has been adapted from (11) and the figure was created in BioRender.com. [file Image_2.jpeg]
